# Enhancement of the apoptotic effects of Arctii Fructus extracts on cancer cells by the enzymatic bioconversion of lignans

**DOI:** 10.1002/fsn3.1336

**Published:** 2020-04-08

**Authors:** Jung‐Eun Kim, Nam Hyouck Lee, Young Ho Kim, Young Eon Kim, Tae‐Gyu Lim, Kyung‐Mo Song

**Affiliations:** ^1^ Research Group of Food Processing Korea Food Research Institute Wanju‐gun Korea; ^2^ SME Solution Center Korea Food Research Institute Wanju‐gun Korea; ^3^ Research Group of Traditional Food Korea Food Research Institute Wanju‐gun Korea

**Keywords:** apoptosis, arctigenin, Arctii Fructus, bioconversion, cancer

## Abstract

The fruit of *Arctium lappa* L. (Arctii Fructus) is one of the most popularly used medicinal plant components in Asia. To enhance the functionality of Arctii Fructus extract, a bioconversion method was developed to produce arctigenin from arctiin. Treatment with β‐glucosidase increased the arctigenin content by >5 fold in Arctii Fructus extracts. The bioconversion products enhanced the apoptosis of cancer cells. The cell viabilities of gefitinib‐resistant lung cancer HCC827 (HCC827GR) cells and colon cancer cells (DLD1) were decreased by 40% and 35%, respectively. The bioconversion products also decreased anchorage‐independent growth of cancer cells. In addition, the increase of apoptosis in cancer cells by bioconversion was confirmed by the flow cytometry analysis. These results indicated that arctigenin exerts anticancer effects on lung and colon cancer cells and that Arctii Fructus can potentially function as a chemopreventive agent. In addition, bioconverted Arctii Fructus extract displayed higher anticancer activity than the same levels of purified arctigenin, indicating the advantage of consuming Arctii Fructus itself as a food or medicinal material.

## Graphical abstract

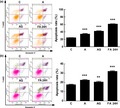

## 1 INTRODUCTION

*Arctium lappa* L. contains various active compounds such as arctiin, arctigenin, tannins, lappaols, and diarctigenin (Chan et al., 2011). These compounds are known to have antioxidant (Wu, Sun, et al., 2014), anti‐inflammatory (Lee & Kim, 2010; Zhao, Wang, & Liu, 2009), antitumor (Awale et al., 2006), and antiproliferative (Ryu, Ahn, Kang, & Han, 1995) activities. Arctigenin and its glucoside arctiin are known as phenylpropanoid dibenzylbutyrolactone lignans (Eich et al., 1996; Wu, Yang, et al., 2014) and can be found in the seeds, stalks, foots, and fruits of *A. lappa* L. (Liu, Chen, Schliemann, & Strack, 2005). The fruit of *A. lappa* L., Arctii Fructus*,* is one of the most popularly used traditional medicinal plant components in Asian countries. The lignans in Arctii Fructus, arctiin and arctigenin, are known to be effective in controlling high blood glucose and diabetes (Wang et al., 2005; Xu et al., 2015). Arctiin is a glucoside form of arctigenin having one glucosyl unit linked with a β‐1,4‐linkage. Many researchers have reported that the aglycone forms of phytochemicals are more active than their glucoside forms because of their effective absorption in the body (Izumi et al., 2000; Walsh et al., 2007; Zubik & Meydani, 2003). Similarly, the aglycone arctigenin is known to have more functionality than the glucoside arctiin (Kim et al., 2010; Wu, Yang, et al., 2014). Because arctigenin is found at much lower levels than arctiin in nature, it is necessary to develop a method to convert arctiin to arctigenin, thereby enhancing production of the more bioactive lignan.

Enzymatic bioconversion has been employed in many conversion processes because of its high economic efficiency, resulting from a high conversion yield, and mild and environmentally friendly conditions (Liu et al., 2014). In this study, we developed a method for the enzymatic bioconversion of arctiin to arctigenin, using β‐glucosidase. β‐glucosidase (E.C. 3.2.1.21) is an enzyme that catalyzes the hydrolysis of β‐1,4‐glycosidic linkages from the nonreducing ends of glycosides. Several previous reports have described enzymatic reactions that can be used to convert arctiin to arctigenin (Jung, Lee, Hyun, & Kim, 2012; Kim et al., 2010; Zhao et al., 2009). Nevertheless, these reactions were not very efficient due to low substrate concentrations and long reaction times, probably caused by the low solubility of arctiin. In the present study, we endeavored to establish an effective bioconversion method using ethanol as the solvent to increase the substrate concentration.

Cancer is one of the leading causes of human death, despite substantial efforts, including the development of new treatments. Thus, continued efforts to discover new therapeutic substances that are more efficacious with fewer side effects are ongoing. Nutraceuticals such as phytochemicals represent a large part of chemopreventive agent. The arctigenin also could induce apoptosis in breast, ovarian, lung, and colon cancer cells (Hosseini & Ghorbani, 2015; Shu, Cheung, Khor, Chen, & Kong, 2010).

In this study, we evaluated the anticancer efficacy of bioconversion products in lung (HCC827GR) and colon (DLD1) cells. The results confirmed the possibility of using Arctii Fructus as a source of anticancer materials, highlighting the possibility of increasing their functionality through enzymatic bioconversion.

## 2 MATERIALS AND METHODS

### 2.1 Materials

Arctii Fructus was purchased from a local market in Seoul, Korea. Arctiin and arctigenin were purchased from Santa Cruz Biotechnology. Cell culture media, including RPMI‐1640 and glucose‐deprived RPMI‐1640, were purchased from Gibco. Fetal bovine serum (FBS), trypsin‐EDTA, and Dulbecco's phosphate‐buffered saline (DPBS) were purchased from Hyclone. *ρ*‐nitrophenol (4‐NP), *ρ*‐nitrophenyl β‐D‐glucopyranoside (4‐NPG), lyophilized β‐glucosidase from almonds, and all other reagents were obtained from Sigma‐Aldrich.

### 2.2 Enzyme assays

The activity of β‐glucosidase against 4‐NPG was measured at 37°C and pH 5.0. The reaction mixture contained 80 μl of 100 mM sodium acetate buffer (pH 5.0) and 80 μl of 5 mM substrate were preincubated at 37°C for 2 min. Hydrolysis was initiated by adding 40 μl of preheated enzyme solution at 37°C. A 50‐μl aliquot was mixed with 100 μl of 1 M sodium carbonate at each time point to stop the reaction. The absorbance of mixture was measured spectrophotometrically at 400 nm using a SpectraMax M2 microplate reader (Molecular Devices). The concentration of 4‐NP was calculated from a standard curve. One unit of enzyme activity was defined as the amount of enzyme that released 1 μmol of 4‐NP per min. The enzyme activity was measured in triplicate.

### 2.3 Thermal and ethanol stability of β‐glucosidase

To determine the thermal and ethanol stability of β‐glucosidase, the enzyme was mixed with ethanol at various concentrations (0%–15%) and incubated at 37°C for 24 hr. The residual activity of incubated enzyme was measured as described above. The activity of enzyme before incubation was considered as 100%.

### 2.4 Enzymatic bioconversion of arctiin and Arctii Fructus extract by β‐glucosidase

For the bioconversion of arctiin, reaction mixtures containing various concentrations of arctiin (5–15 mM) and ethanol (5%–15%), 40 mM sodium acetate buffer (pH 5.0), and β‐glucosidase were incubated at 37°C. For the bioconversion of Arctii Fructus, an extract was prepared as follows. Arctii Fructus was blended into a powder using an HMF‐3100S blender (Hanil Electric Co.). Ten grams of powdered sample was combined with 1,000 ml of 60% ethanol, and the mixture was incubated with stirring at 25°C for 24 hr. The extracts were centrifuged at 2,800 *g* and 4°C for 15 min, and the supernatants were filtered through Whatman No. 4 filter paper (Whatman International Ltd.) and freeze‐dried. The extract was dissolved in ethanol and processed in bioconversion reactions. A reaction mixture containing 10 mg/ml extract and 5% ethanol was incubated at 37°C with 40 mM sodium acetate buffer (pH 5.0) and 5 U/ml β‐glucosidase. The reaction was terminated by boiling for 5 min.

### 2.5 Analysis of the bioconversion products using high‐performance liquid chromatography (HPLC)

The bioconversion products were analyzed by HPLC using a Dionex Ultimate 3000 system (Thermo Fisher Scientific) consisting of an autosampler, a column oven, a quaternary pump with a built‐in solvent degasser, and the Chromeleon software program. The components were eluted using an Xterra RP18 column (250 × 4.6 mm; Waters Co.) at 40°C with a flow rate of 0.8 ml/min and detected at 280 nm using a UV detector. Mobile phase A and B were 0.2% phosphoric acid and 100% acetonitrile, respectively. The gradient program was as follows: a linear gradient of B from 20% to 60% for 15 min, a linear decrease to 20% mobile phase B for 1 min, followed by a hold at 20% mobile phase B for 9 min. The concentrations of arctiin and arctigenin were calculated based on the peak areas of standard substances. The bioconversion yield was calculated using the following equation:

$$\text{Bioconversion}\;\text{yield}\;\text{of}\;\text{arctiin}=\frac{[\text{arctigenin]}}{\left[\text{arctiin}\right]+\left[\text{arctigenin}\right]}*100$$

### 2.6 Cell culture and cell‐viability assays

Human colorectal adenocarcinoma DLD1 cells were purchased from the American Type Culture Collection (ATCC). HCC827GR (gefitinib‐resistant lung cancer) cells were kindly provided by Dr. Pasi A. Jänne at the Department of Medical Oncology, Dana‐Farber Cancer Institute, Boston, MA (Engelman et al., 2007). Colon cancer DLD1 cells and gefitinib‐resistant lung cancer HCC827 cells (HCC827GR) were grown in RPMI‐1640 supplemented with 10% FBS. All cells were maintained as monolayer cultures at 37°C in an incubator with 5% CO_2_. DLD1 and HCC827GR cells were seeded in a 96‐well plate at a density of 5 × 10^4^ and 1 × 10^5^ cells per each well, respectively. After a 24‐hr incubation, various concentrations of arctiin, arctigenin, and bioconversion samples in glucose‐deprived RPMI‐1640 were added and incubated for another 24 hr. Cell viability was determined by performing 3‐(4,5‐dimethylthiazol‐2‐yl)‐5‐(3‐carboxymethoxyphenyl)‐2‐(4‐sulfophenyl)‐2H‐tetrazolium (MTS) assays using the CellTiter 96 AQueous MTS Reagent (Promega). Twenty microliters of MTS solution was added to each well, and the cells were further incubated at 37°C for 30 min in an atmosphere containing 5% CO_2_. Next, the absorbance was measured using a microplate reader (Molecular Devices) at 490 nm and at 650 nm as the reference absorbance.

### 2.7 Assaying anchorage‐independent growth in soft agar

Soft‐agar assays were performed to evaluate anchorage‐independent growth of cancer cells. Three milliliters of 0.6% bottom agarose were placed in separate wells of 6‐well plates with or without arctiin, arctigenin, Arctii Fructus extract, or bioconversion samples. After solidification of the bottom agarose, 2.4 × 10^4^ cells were resuspended in 0.3% top agarose, with or without sample, and 1 ml of the top agarose containing 8 × 10^3^ cells was placed on the bottom agarose. The cells were incubated at 37°C in a 5% CO_2_ incubator for 3 weeks. The colonies were detected using an Eclipse Ti‐S microscope (Nikon) and photographed using a MetaMorph software program (Molecular Devices).

### 2.8 Analysis of apoptosis by an Annexin V‐PE and 7‐aminoactinomycin D (7‐AAD) staining assay by flow cytometry

HCC827GR and DLD1 cells were grown in RPMI‐1640 supplemented with 10% FBS. All cells were maintained as monolayer cultures at 37°C in an incubator with 5% CO_2_. The cells were seeded in 10 cm plates at a density of 25 × 10^4^ cells/dish. After a 24‐hr incubation, 0.5 μM arctiin, 0.5 μM arctigenin, 1 μg/ml Arctii Fructus extract, or 1 μg/ml bioconverted sample was added to the plates in glucose‐deprived RPMI‐1640 and incubated for another 24 hr. Floating and adherent cells were collected after trypsinization with trypsin‐EDTA solution. The cells were prepared for analysis with the PE Annexin V Apoptosis Detection Kit (BD Biosciences), following the manufacturer's protocol. Briefly, the cells were washed twice with cold PBS, the cell pellets were suspended in ice‐cold 1× binding buffer at a density of 1 × 10^6^ cells/ml, and then, 100 μl of each cell suspension was incubated with 5 μl of Annexin V‐PE and 7‐AAD for 15 min in the dark at room temperature. The treated cells were diluted 5 times and analyzed by flow cytometry (CytoFLEX, Beckman Coulter, Inc.) within 1 hr of staining.

## 3 RESULTS AND DISCUSSION

### 3.1 Stability of β‐glucosidase enzymes

**Figure 1:**
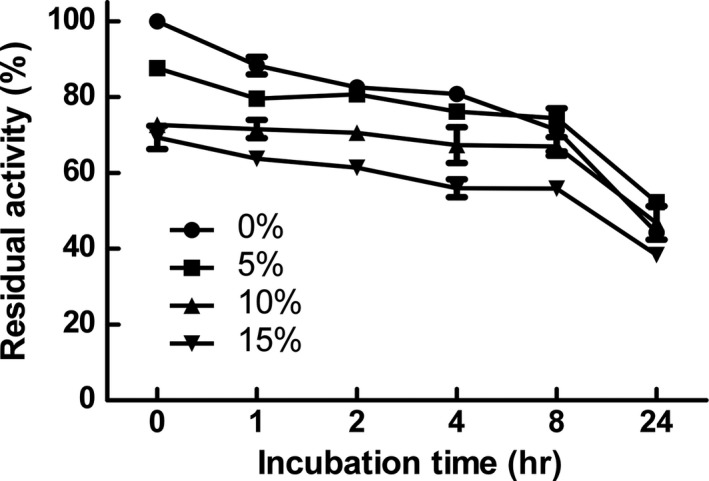
Ethanol stability of β‐glucosidase. The enzyme was incubated at 37°C and pH 5.0 with various concentrations of ethanol, and the residual activity was measured at several time points. Reaction mixtures contained various concentrations of ethanol (circle, 0% ethanol; square, 5% ethanol; triangle, 10% ethanol; inverted triangle, 15% ethanol). The data shown represent the mean percentage levels ± the standard deviation (*SD*)

In this study, ethanol was used as a solvent to dissolve arctiin because of the low solubility of arctiin in water, as well as the low toxicity and cost of ethanol. The stability of β‐glucosidase was determined by measuring its residual activity after incubation in various concentrations of ethanol (up to 15%) (Figure 1). The residual activities of β‐glucosidase decreased with increasing incubation times. When the starting enzyme activity was set at 100%, the residual activity after 24 hr was >40% at all ethanol concentrations. Similarly, several β‐glucosidases showed stability in ethanol. For example, β‐glucosidase from *Aspergillus niger* was stable at ethanol concentration of <30% (Zhao, Zhou, Li, Fan, & You, 2013), and β‐glucosidase from *Myceliophthora thermophile* retained its original activity until 6 hr after incubation in 50% ethanol (Karnaouri, Topakas, Paschos, Taouki, & Christakopoulos, 2013). In addition, a mutagenized β‐glucosidase from a marine microbe converted isoflavone to its aglycone in 10 min in the presence of 18% ethanol with >98% of residual activity (Fang et al., 2016). These findings revealed that some β‐glucosidases might be potentially used for bioconversion processes, including the arctiin bioconversion. According to the results, β‐glucosidase from almonds retained >60% of activity after an 8‐hr incubation; thus, arctiin bioconversion was conducted within 8 hr.

### 3.2 Enzymatic bioconversion of arctiin and Arctii Fructus extract

Bioconversion conditions were evaluated at pH 5.0, 37°C, 5–15 mM arctiin, and 5%–15% ethanol considering the stability of enzyme. The reaction mixtures were analyzed by HPLC to monitor arctigenin production. The concentrations of arctiin and arctigenin after enzymatic bioconversion were calculated from their peak areas, as were their bioconversion yields. Five millimolar arctiin was converted to arctigenin with almost 100% efficiency after an 8‐hr reaction, and 10 mM arctiin was bioconverted by >70% after a 7‐hr reaction (data not shown). In contrast, the bioconversion of 15 mM arctiin to arctigenin did not exceed 40% during an 8‐hr reaction (data not shown).

**Figure 2:**
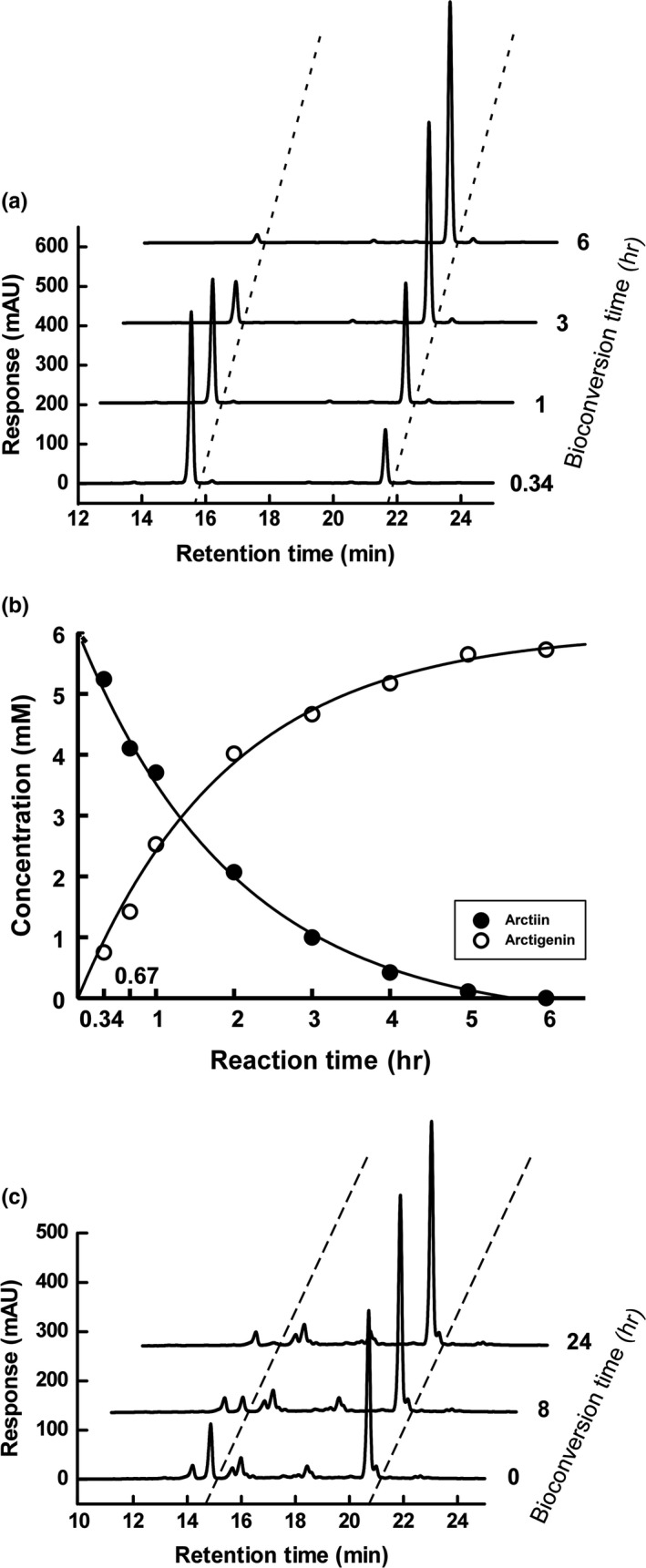
Analysis of 5 mM arctiin and 10 mg/ml of the Arctii Fructus extract bioconversion mixture using high‐performance liquid chromatography (HPLC). The HPLC chromatograms show (a) the arctiin bioconversion products analyzed at each time point, (b) changes of arctiin and arctigenin contents during bioconversion (closed circle, arctiin; open circle, arctigenin), and (c) Arctii Fructus extract bioconversion products

The bioconversion result of 5 mM arctiin in 5% ethanol solution was shown in Figure 2a,b. The bioconversion yield was >90% after a 4‐hr reaction, and the conversion was completed after 6 hr reaction (100%). Bioconversion of the Arctii Fructus extract was conducted under the same conditions, and the results are shown in Table 1 and Figure 2c. The ratio of arctiin and arctigenin from Arctii Fructus extract increased fivefold after a 24‐hr reaction. These results reflected improved bioconversion conditions, compared with previous results. Kim et al. (2010) reported the complete conversion of arctiin to arctigenin by fermentation of Arctii Fructus extract with β‐glucosidase, although that took 72 hr. In another previous study, the initial arctigenin content in Arctii Fructus increased by approximately 4.8‐fold after enzymatic hydrolysis for 24 hr (Liu et al., 2014), but the extracts were dissolved in 5% methanol, which is an undesirable solvent for food processing.

**Table 1:** Contents of arctiin and arctigenin, and the ratio of arctiin to arctigenin in Arctii Fructus extract during bioconversion

Reaction time (hr)	Arctiin (mM)	Arctigenin (mM)	Ratio
0	1.9	4.7	1:2.4
2	1.1	2.9	1:2.6
4	1.0	3.3	1:3.5
6	0.9	4.0	1:4.3
8	1.1	5.6	1:5.2
24	0.8	9.6	1:11.9

### 3.3 Apoptotic effects of bioconversion products on cancer cells

#### 3.3.1 Cell viability of cancer cells

**Figure 3:**
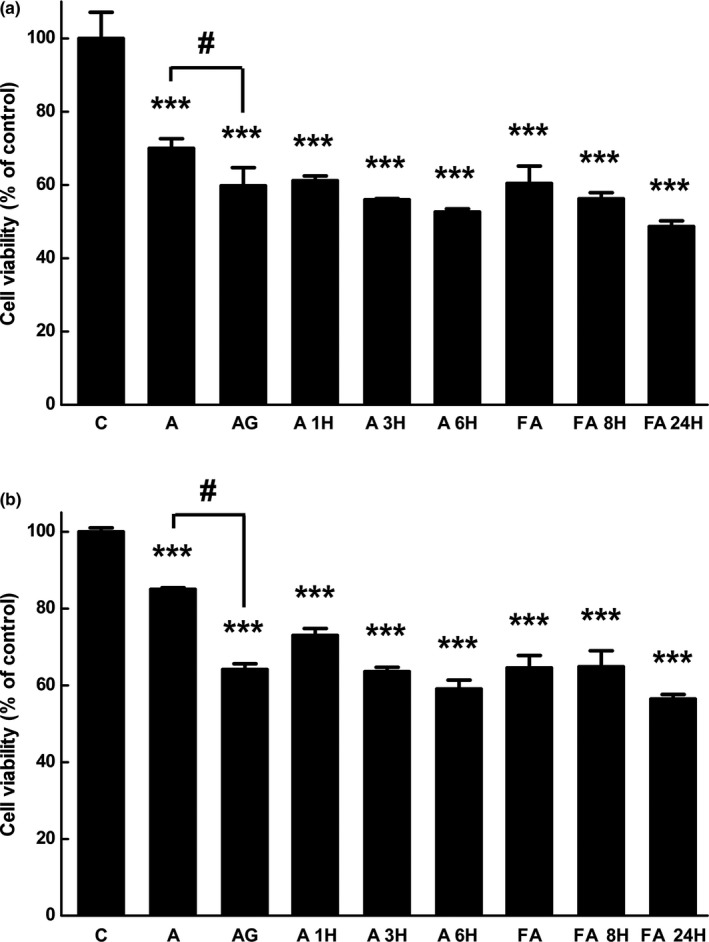
Effects of arctiin, arctigenin, and bioconversion products of Arctii Fructus extract on the cell viability of (a) gefitinib‐resistant lung cancer cells (HCC827GR) and (b) colon cancer cells (DLD1). The cell viabilities of HCC827GR and DLD1 cells treated with 0.5 μM arctiin, 0.5 μM arctigenin, 1 μg/ml Arctii Fructus extract, or 1 μg/ml bioconversion products were detected by 3‐(4,5‐dimethylthiazol‐2‐yl)‐5‐(3‐carboxymethoxyphenyl)‐2‐(4‐sulfophenyl)‐2H‐tetrazolium (MTS) assays. The data shown represent the mean percentages of 3 different experiments ± *SD*. #, statistically significant difference between the indicated constructs. **p < .01, ***p < .001, compared with the control by analysis of variance testing

The 1‐, 3‐, and 6‐hr reaction mixtures with arctiin and the 0‐, 8‐, and 24‐hr reaction mixtures with Arctii Fructus extract were selected for subsequent experiments, given their similarities in arctigenin concentration. At first, the apoptotic activity of arctiin and arctigenin was tested using 4 different lung cancer cell lines (HCC827, HCC827GR, H460, and H1299) and colon cancer cell lines (HCT116, HCT15, HT29, and DLD1). Results showed that arctigenin has the highest apoptotic activity toward HCC827GR and DLD1 (data not shown). Thus, further experiments were conducted using these cell lines. First, the effects of different samples on cell viability were determined (Figure 3a, HCC827GR; 3b, DLD1). The concentrations of extract and its bioconversion products were determined at 1 μg/ml, as this value was suitable for presenting the bioconversion effect. At higher and lower concentrations, there were no significant differences between the samples because of their relatively high and low apoptotic activities, respectively (data not shown). Additionally, 0.5 μM of arctiin and arctigenin, which were similar concentrations to those in extract, was treated independently and compared with extracts. In HCC827GR cells, arctiin and arctigenin showed 30% and 40% decreases in absorbance, respectively, compared to untreated control cells. The Arctii Fructus extract showed a 40% decrease of cell viability. The viability of cells treated with bioconversion samples decreased as the bioconversion reaction time increased and were reduced 48% and 52% by a 6‐hr reactant of arctiin and 24‐hr reactant of Arctii Fructus, respectively. The results obtained with DLD1 cells were similar. Arctiin and arctigenin treatment reduced the cell viability by approximately 15% and 35%, respectively. The Arctii Fructus extract caused a 35% decrease in cell viability, and the viability of cells exposed to bioconverted samples decreased as the bioconversion reaction time increased. Finally, the cell viabilities were decreased to 41% and 44% by treatment of 6 hr arctiin and 24 hr Arctii Fructus reaction mixtures, respectively.

The cell viability of 2 types of cancer cells decreased with increasing bioconversion times due to the increased arctigenin concentrations, and the cancer cells were susceptible to a low concentration of arctigenin. The anticancer activities of arctigenin have been studied by many research groups. Han et al. (2016) reported that arctigenin decreased the cell viability of metastatic colon cancer cells by inducing apoptosis and prevented metastasis to the lungs. According to research by Susanti, Iwasaki, Inafuku, Taira, and Oku (2013), arctigenin decreased the cell viability and induced apoptosis in lung adenocarcinoma cell. Interestingly, the results generated in this study showed that arctigenin was highly effective in inducing cell death in gefitinib‐resistant lung cancer cells at a very low concentration (0.5 μM).

#### 3.3.2 Anchorage‐independent performed cell growth in soft agar

**Figure 4:**
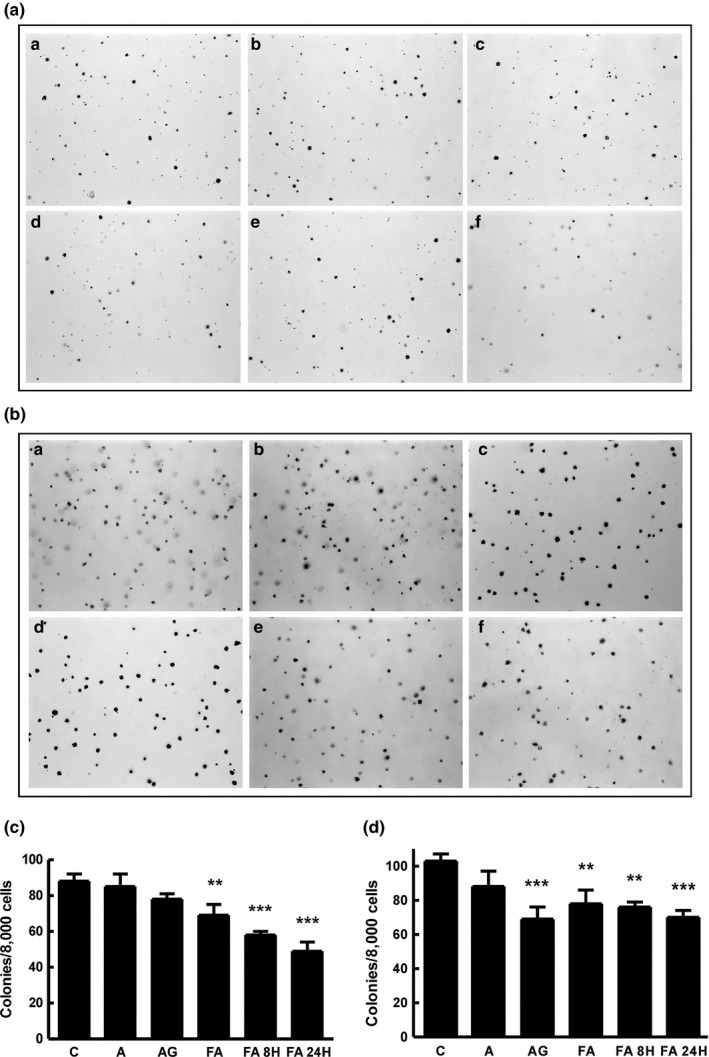
The cell growth inhibitory effects of samples were evaluated by measuring anchorage‐independent growth in soft agar (a, HCC827GR; b, DLD1). The cells were detached and resuspended in soft agar containing samples and photographed after 3 weeks. The representative fields were photographed at ×4 magnification (a, control; b, arctiin; c, arctigenin; d, 0‐hr bioconverted Arctii Fructus extract; e, 8‐hr bioconverted Arctii Fructus extract; f, 24‐hr bioconverted Arctii Fructus extract). The data represent the mean percentages of 3 different experiments ± *SD*. **p < .01, ***p < .001, compared with the control by analysis of variance testing

Several experiments were performed to determine whether the decrease in cell viability was due to apoptosis. First, we determined whether bioconversion products can inhibit anchorage‐independent growth in soft agar. Anchorage‐independent growth is a key characteristic of metastatic cancer cells. Figure 4a,b show that HCC827GR and DLD1 cells could survive and grow under anchorage‐independent growth conditions and that cell growth was reduced by treatment with 0.5 μM arctiin, 0.5 μM arctigenin, 1 μg/ml Arctii Fructus extract, or 1 μg/ml bioconversion sample. Whereas arctiin reduced HCC827GR cell growth by 12%, the bioconversion sample reduced cell growth by up to 45% (24‐hr bioconverted Arctii Fructus extract). It was previously reported that butein suppressed anchorage‐independent growth by 40% and 50% in HCC827 and HCC827GR cells, respectively, with a minimum butein concentration of 12.5 μM (Jung et al., 2015). Also, 40 μM isoliquiritigenin suppressed anchorage‐independent growth by 40% in HCC827GR cells (Jung et al., 2014). These findings indicate that Arctii Fructus is more effective than these already known anticancer substances and support a role for arctigenin in suppressing cancer cell growth. Furthermore, treatment of cells with the 24 hr bioconversion product of the extract inhibits cancer cell survival much more efficiently than similar concentrations of arctiin and arctigenin. This is possibly due to a synergistic effect of the various bioactive compounds found in the extract, including arctigenin. A similar result was reported for cranberry extract where the total extract showed a higher anticancer effect than that of individual compounds (Seeram, Adams, Hardy, & Heber, 2004). The bioconversion of various components including arctiin in the Arctii Fructus extract was probably responsible for increasing the inhibitory effect on cancer cells in a synergistic manner.

#### 3.3.3 Cellular apoptosis analysis by flow cytometry

**Figure 5:**
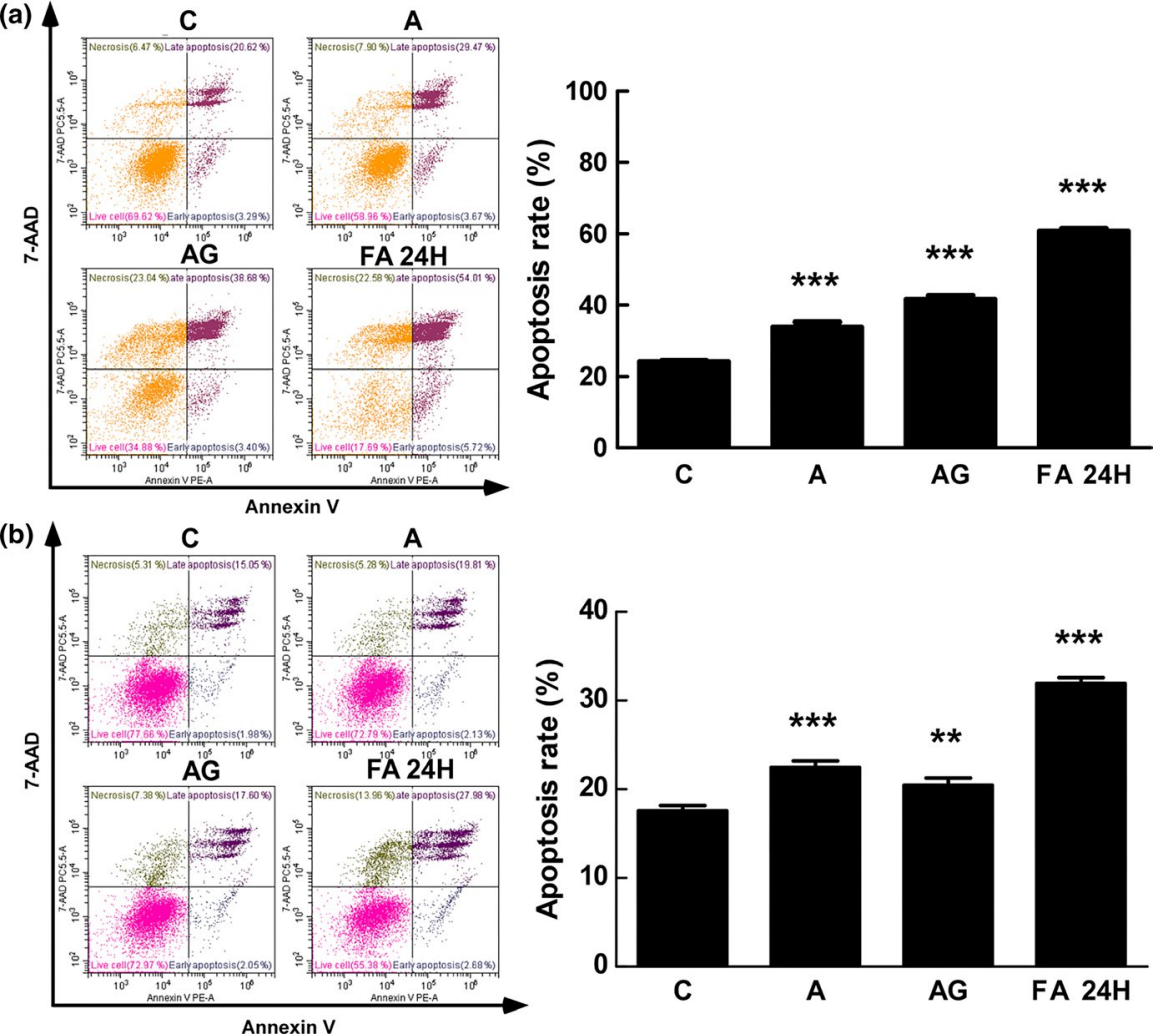
Analysis of apoptosis in cancer cells by flow cytometry (a, HCC827GR; b, DLD1). HCC827GR and DLD1 cells were treated with or without the bioconverted samples for 24 hr. The patterns observed after double staining of annexin V‐PE and 7‐AAD showed annexin V‐positive, 7‐AAD‐negative events (early apoptosis cells); double‐positive events (late apoptosis cells); annexin V‐negative, 7‐AAD‐positive events (necrosis cells); and double‐negative events (live cells). The data represent the mean percentages of 3 different experiments ± *SD*. **p < .01, ***p < .001, compared with the control by analysis of variance testing. C, control; A, arctiin; AG, arctigenin; FA 24H, 24‐hr bioconverted Arctii Fructus extract

To investigate whether arctigenin induced cell death via apoptosis, HCC827GR and DLD1 cells were treated with 0.5 μM arctiin, 0.5 μM arctigenin, 1 μg/ml Arctii Fructus extract, or 1 μg/ml bioconversion sample for 24 hr, after which apoptosis was studied using the PE Annexin V Apoptosis Detection Kit. The rate of apoptosis was detected by flow cytometry after double staining of annexin V‐PE and 7‐AAD (Figure 5a, HCC827GR; 5b, DLD1). The apoptosis rate in HCC827GR cells was 24%, and exposure to arctiin and arctigenin increased the apoptosis rate 33% and 41%, respectively. Interestingly, treatment with the Arctii Fructus bioconversion sample increased the apoptosis rate to 60%. The concentration at which butein induced 50% apoptosis was 50 μM in HCC827GR cells (Jung et al., 2015), and 40 μM isoliquiritigenin induced 20% apoptosis in the same cell type (Jung et al., 2014). In this study, the concentration at which arctigenin induced 40% apoptosis was 0.5 μM, and the concentration at which the Arctii Fructus bioconversion sample showed 60% apoptosis was 0.9 μM arctigenin. Therefore, arctigenin and Arctii Fructus were more effective in promoting apoptosis than butein and isoliquiritigenin. The apoptosis rate in DLD1 cell was 17%, and exposure to arctiin, arctigenin, and the Arctii Fructus bioconversion sample increased the apoptosis rate 22%, 20%, and 31%, respectively. Results revealed that similar to arctiin, arctigenin exhibits low apoptotic activity, even though it exhibits a high inhibitory effect on the proliferation of DLD1 cells (Figure 3b). This effect might be due to the higher necrosis induced by arctigenin treatment (Figure 5b). Interestingly, the 24 hr bioconversion product of Arctii Fructus extract showed greater apoptotic activity than both arctiin and arctigenin. This is probably due to other components that are present in the bioconverted extract or due to the synergistic effect of these compounds. These results indicated that Arctii Fructus bioconversion product induced apoptosis in both DLD1 cells and HCC827GR cells. However, treatment with arctigenin alone was effective in HCC827GR cells, but not in DLD1 cells. This indicates that the anticancer activity of the bioconversion product of Arctii Fructus extract can be attributed to arctigenin along with other compounds present in the extract that act in a synergistic manner.

In this study, we confirmed the anticancer activity of arctigenin and Arctii Fructus extract toward lung and colon cancer cells. The apoptotic effects of arctigenin have been also identified toward other types of cancer cells. For example, arctigenin‐based suppression of the iNOS/NO/STAT3/survivin signaling pathway inhibited cell growth and induced caspase‐3‐dependent apoptosis in ovarian cancer cells (Huang et al., 2014). Arctigenin also reduced the cell viability and induced apoptosis by directly activating the mitochondrial pathway, reducing phospho‐ERK1/2 expression, and inducing phospho‐p38 levels in bladder cancer T24 cells (Yang et al., 2012). Moreover, arctigenin treatment induced upregulation of reactive oxygen species, p38 MAPK, and H3K9 trimethylation and downregulation of Bcl‐2, which inhibited cell growth and caused apoptosis in breast cancer MDA‐MB‐231 cells (Hsieh et al., 2014). Furthermore, arctigenin enhanced apoptosis of cancer cells in response to cisplatin, a chemotherapeutic drug (Wang, Jin, & Wang, 2014; Yao et al., 2011). In particular, the extract of *A. lappa* L. and arctigenin showed cytotoxicity toward lung, liver, and stomach cancer cells, whereas no cytotoxicity was observed toward several types of normal cells (Susanti et al., 2012). Based on these studies and our results, it is anticipated that arctigenin may be capable of synergizing with chemotherapeutic drugs and can possibly serve as a cancer chemopreventive agent.

## 4 CONCLUSION

In the present study, we employed environmentally friendly methods using enzymes and finally obtained a Arctii Fructus extract containing high concentration of arctigenin. This bioconverted Arctii Fructus extracts revealed anticancer effects on lung and colon cancer cells, which were confirmed by studying the induction of apoptosis. Therefore, Arctii Fructus has the potential to function as a chemopreventive agent. Furthermore, the extract displayed a higher anticancer effect than did single treatment of the same amount of arctigenin that was contained within the extracts. This appears to be a synergistic effect of the components included in mixture, which means that the extract is useful as a functional food material.

## CONFLICT OF INTEREST

We declare that we have no conflict of interest.

## ETHICAL APPROVAL

This study does not involve any human or animal testing.
